# Carcinoid Heart Disease: Starting From Heart Failure

**DOI:** 10.1177/2324709617713511

**Published:** 2017-06-08

**Authors:** Nicole Bertin, Serena Favretto, Francesco Pelizzo, Lucio Mos, Franco Pertoldi, Olga Vriz

**Affiliations:** 1 University of Udine, Udine, Italy; 2 University of Eastern Piedmont, Novara, Italy; 3 San Antonio Hospital, San Daniele del Friuli, Udine, Italy

**Keywords:** neuroendocrine tumor, carcinoid syndrome, carcinoid heart disease, tricuspid regurgitation

## Abstract

Carcinoid syndrome is the constellation of symptoms mediated by humoral factors produced by some carcinoid tumors. It consists primarily of vasomotor symptoms, gastrointestinal hypermotility, hypotension, and bronchospasm, due to the production and release of vasoactive substances. Carcinoid heart disease occurs in more than 50% of patients with carcinoid syndrome; in some cases, it represents the initial manifestation of the disease. We report the case of a 75-year-old woman with a metastatic neuroendocrine tumor admitted to the emergency room for fatigue and heart failure. Transthoracic echocardiography showed severe tricuspid and pulmonic regurgitation suggesting carcinoid heart disease. A hypervascular retroperitoneal mass was found on abdominal computed tomography, which seemed to arise from the mesenteric artery, anteriorly to the abdominal aorta. Unfortunately, our patient was neither a candidate for mass resection nor for cardiac surgery due to advanced metastatic disease and poor clinical condition. Additionally, we performed a systematic literature review of carcinoid heart disease focusing on typical echocardiographic findings.

## Introduction

Carcinoid syndrome (CS) is a clinical characteristic of well-differentiated neuroendocrine tumors (NETs), particularly seen in those arising from the midgut (ie, small intestine, appendix, and proximal colon), and less commonly from the lung and the pancreas.^1^ It is due to the production and secretion of a variety of mediators, including serotonin, 5-hydroxytryptamine, 5-hydroxytryptophan, histamine, tachykinins, bradykinin, and prostaglandins, although the precise pathogenesis remains elusive. CS usually occurs in patients with metastatic lesions to the liver, due to the lack of hepatic inactivation of these mediators.

The most common manifestations of CS are vasomotor symptoms (flushing), hypotension, secretory diarrhea, abdominal cramps, and bronchospasm. This syndrome can lead to clinically severe complications caused by tissue fibrotic degeneration, such as carcinoid heart disease (CHD), mesenteric and retroperitoneal fibrosis, and debilitating diarrhea.^2^

CHD is a well-recognized complication of CS and may be the initial presentation of this syndrome in up to 20% of patients; it represents a major cause of morbidity and mortality in patients with CS.^3,4^

## Case Report

A 75-year-old woman presented to the emergency department with fatigue and worsening dyspnea. She complained of chronic diarrhea and weight loss (about 15 kg) in the last 6 months but no flushing.

On physical exam, blood pressure was 164/85 mm Hg, heart rate was 78 bpm (sinus rhythm), peripheral capillary oxygen saturation was 90% with normal respiratory rate, and body mass index was 32 kg/m^2^. She had no fever.

Clinical pathological findings included bibasilar reduction of pulmonary vesicular sounds compatible with bilateral pleural effusion, jugular vein distention, and edema in both legs. On cardiac auscultation, a grade 3/6 pansystolic murmur was heard at the left lower sternal border. There was abdominal distension without signs of peritonitis or shifting dullness.

Arterial blood gas analysis showed mild hypoxemia with normocapnia and moderate hypokalemia with normal anion gap. The electrocardiogram was normal.

She was admitted to the general internal medicine unit for further evaluation.

Blood tests showed normal blood count, a mild increase in inflammatory markers, and cholestasis. A fecal immunochemical test yielded normal results except for elevated levels of fecal calprotectin.

**Figure 1.**
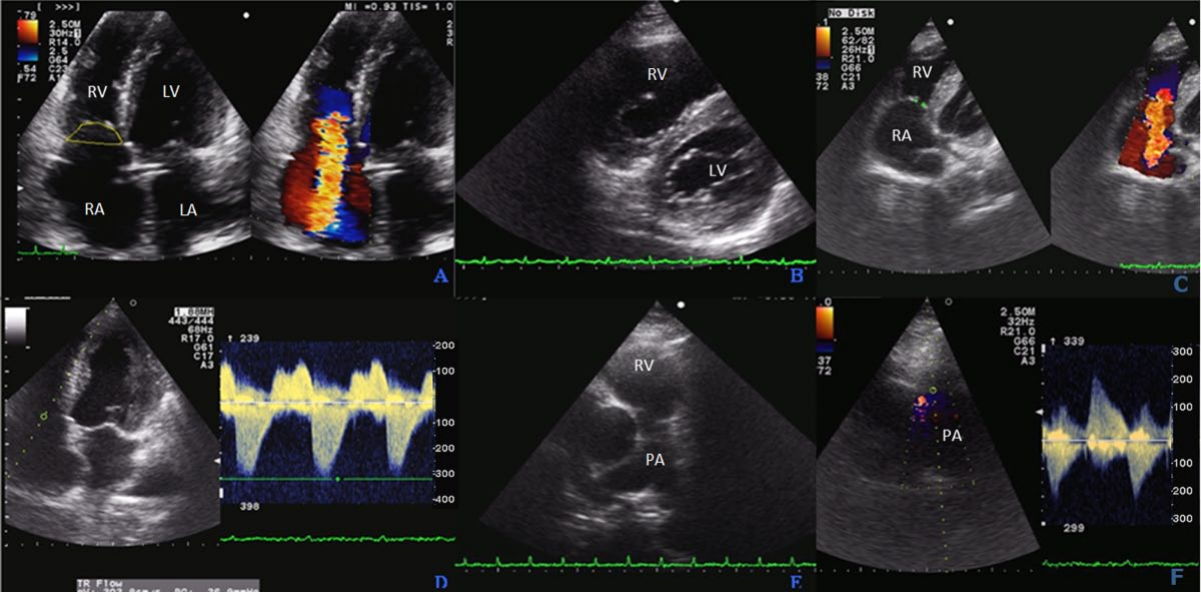
Echocardiographic findings: (A) Four-chamber view showing severe tricuspid regurgitation into a dilated right atrium. On the left, tenting area. (B) Short-axis view showing “D-shaped” left ventricular morphology as sign of right ventricular overload. (C) Tricuspid valve detail showing thickened and retracted valve leaflets failing to coapt and resulting in constant “semi-open” position. (D) Continuous wave (CW) Doppler of the tricuspid valve confirming severe regurgitation. (E) Pulmonary valve with thickening of pulmonary cusps and dilated pulmonary trunk. (F) CW Doppler of the pulmonary valve showing moderate valve stenosis and severe regurgitation with pulmonary hypertension (acceleration time 80 ms). RA, right atrium; RV, right ventricle; LA, left atrium; LV, left ventricle; PA, pulmonary artery.

Abdominal ultrasound was nondiagnostic due to intestinal bloating and visceral obesity; therefore, computed tomography (CT) of the abdomen was performed (Figure 1), showing a hypervascular retroperitoneal mass (8.5 × 11 × 6.5 cm), which seemed to arise from the mesenteric artery, anteriorly to the abdominal aorta. Two hepatic nodules (2 and 3 cm, respectively, compatible with secondary lesions) and multiple adenopathy were also demonstrated.

During hospitalization, the patient developed worsening respiratory failure with episodes of respiratory distress with bronchospasm; spiral CT angiography was then performed, which ruled out pulmonary embolism or pulmonary lesions.

**Figure 2.**
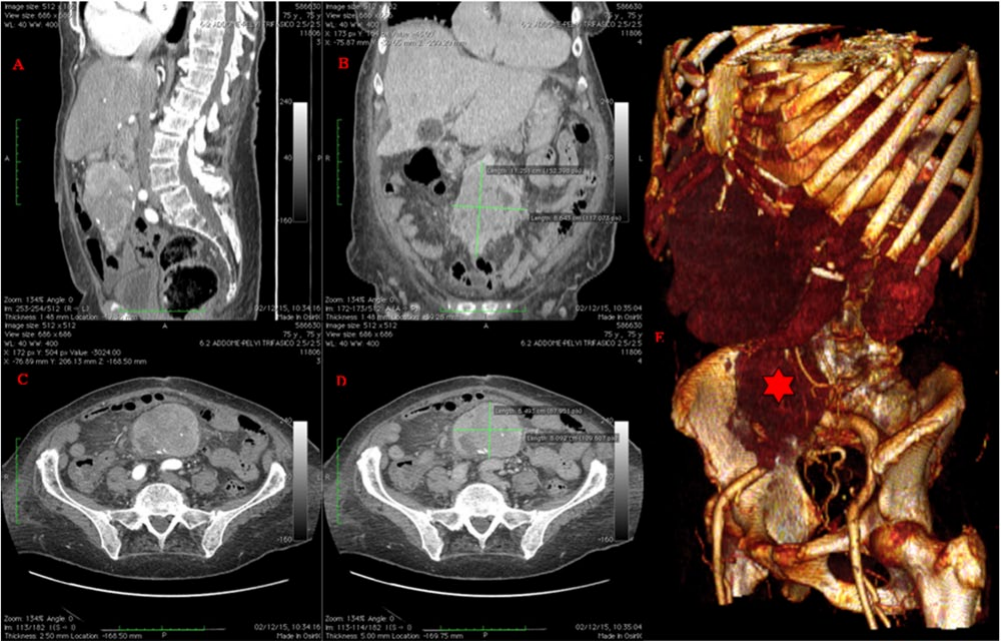
Computed tomography (CT) imaging. (A) Sagittal CT scan (late arterial phase, 35 seconds after bolus-tracking) showing a hypervascular tumor. (B) Coronal CT scan view (late portal phase at 70 seconds) showing slow washout of the primary lesion. (C) Axial CT scan view (arterial phase) confirming vascular enhancement and showing interaction with blood vessels. (D) Axial CT scan view (portal phase). (E) 3D volume rendering reconstruction: the neuroendocrine tumor (red star) is shown.

The patient underwent transthoracic echocardiography, which showed severe tricuspid regurgitation, as demonstrated by increased coaptation depth (a measure of the maximal distance from the leaflet tips to the annular plane) and tenting area (area between the annulus plane and tricuspid leaflets in early systole; Figure 2A and D), and severe pulmonary regurgitation (Figure 2F) with mild to moderate transpulmonary gradient; the maximum velocity of the tricuspidal regurgitant jet was 303.8 cm/s. The pulmonary and tricuspid valves appeared thickened and partially retracted (Figure 2C and E). A flat interventricular septum and a D-shaped left ventricle were demonstrated (Figure 2B), consistent with right ventricular overload and increased right ventricular diastolic pressure. The peak pulmonary artery velocity was 220 cm/s, corresponding to a diastolic pulmonary pressure of 19.4 mm Hg, to which was added the right atrium pressure of 15 mm Hg, resulting in a final diastolic pulmonary pressure of 34.4 mm Hg. The left-sided valves were normal in appearance and function. These findings, along with extremely elevated serum levels of chromogranin A (>4016 ng/mL, normal value = 20-185 ng/mL), raised the suspicion of an NET originating in the small intestine with secondary spread to the mesentery, liver, and lymph nodes, with associated CS with heart involvement.

**Figure 3.**
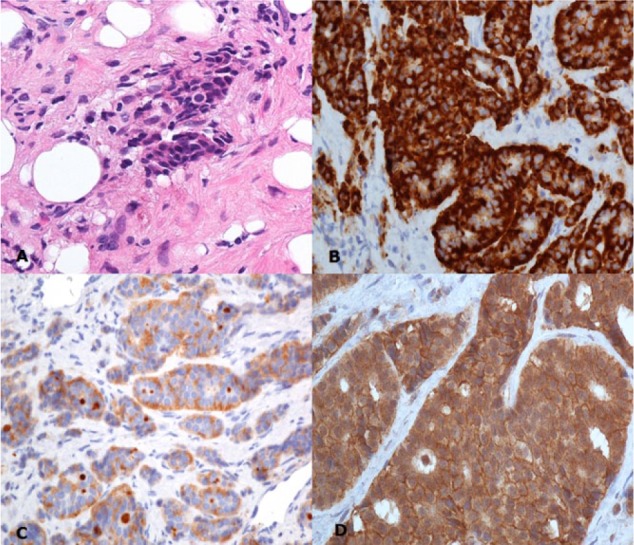
Abdominal mass biopsy confirming diagnosis of a well-differentiated neuroendocrine tumor (A) and immunohistochemical stains for chromogranin (B), synaptophysin (C), and somatostatin receptor type 2 (D).

A laparoscopic biopsy confirmed the diagnosis of a well-differentiated NET (Figure 3), but unfortunately the mass was inoperable due to vascular invasion. Owing to the progressive nature of the malignancy, the patient was not deemed to be a candidate for cardiac surgery.

Medical treatment with somatostatin analogues was started to reduce hormone-related symptoms, but the patient died after a few months of medical therapy.

## Discussion

NETs are heterogeneous neoplasms arising from neuroendocrine precursor cells of different tissues.

The term “carcinoid” is generally applied to well-differentiated NETs originating from the digestive tract, the lungs, and, more rarely, from primary sites such as the kidneys or ovaries.

CS is caused by the intermittent release of serotonin and other vasoactive substances from the tumor into the systemic circulation. It is frequently a sign of disseminated disease in patients with a primary tumor in the midgut or the bronchial system and generally consists of episodes of flushing, secretory diarrhea, and bronchospasm.^5^ According to the US Surveillance, Epidemiology, and End Results Program, the incidence of NETs from 2000 to 2004 was 5 cases per 100 000 individuals.^6^

CHD, also known as Hedinger’s syndrome, is estimated to occur in 20% to 50% of patients with CS.^7,8^ Other studies report cardiac lesions of various degrees in up to 60% of patients with CS.^9^ Typically, fibrous plaque-like and endocardial thickening cause retraction and fixation of the right-sided heart valves, often leading to severe right heart failure.^1,10-13^ CHD occurs as a consequence of chronic exposure of the cardiac endothelium to serotonin, usually after a mean period of 1.5 years. Simultaneous involvement of both valves of the right heart strongly suggests CHD as the likely diagnosis, representing a pathognomonic sign. Serotonin is probably the main but not the sole determinant of cardiac involvement in NETs, and other mediators detected in the endocardial plaques of CHD patients, such as activin A (a member of the TGF-β family), were found to play a role.

Monitoring of progression of cardiac involvement is important as CHD can dramatically affect long-term survival, and valve replacement is the only definitive treatment option.^14^

CHD frequently occurs in patients with NETs of the small bowel (72%), followed by NETs originating from the lungs, large bowel, pancreas, appendix, or ovaries. Nonetheless, in approximately 18% of cases the primary tumor site cannot be identified.^10^ A slight male preponderance (60%) has been reported, with a mean age at diagnosis ranging from 56 to 63 years.

Echocardiography is the primary imaging technique for diagnosing CHD. Transthoracic echocardiography can reveal thickening of the valve leaflets, chords, and papillary muscles. Tricuspid valve involvement is reported in up to 90% of patients. Valve leaflets typically appear thickened and become fixed and retracted in semi-open position with disease progression, resulting in valve regurgitation with concomitant stenosis of variable degree.^15,16^ Severe tricuspid regurgitation leads to right ventricular volume overload followed by right ventricular dilatation in 90% of patients; right atrial dilatation is observed in all patients. Involvement of the pulmonary valve may also occur, though less frequently (49% to 69% of patients). Thickening of the pulmonic valve cusps is usually observed, as carcinoid plaque deposition results in adherence of the leaflets to the underlying endocardium, causing valve stenosis and regurgitation and progressive dilatation of the right-sided cardiac chambers.^15^

Left-sided valvular disease is less common. In a retrospective series, it was mainly observed in patients with patent foramen ovale (87%) and, less frequently, in patients with bronchial carcinoids (13%).^15^

The European Neuroendocrine Tumor Society consensus guidelines state that annual echocardiography is mandatory as part of the routine surveillance of patients with CHD.^17^ These guidelines also suggest a “bubble study” to exclude patent foramen ovale.

Advanced CHD can be easily identified by transthoracic echocardiography. However, in the early stages of the disease, the diagnosis may be challenging, and current American and European guidelines do not address the issue of how to best manage patients with asymptomatic or minimally symptomatic CHD.^17-21^

Other imaging modalities may be useful to establish the diagnosis and to assess disease severity. Cardiac magnetic resonance overcomes the issue of suboptimal visualization of the right-sided heart valves and has the advantage of enabling accurate quantification of regurgitant volumes and right ventricular ejection fraction,^22,23^ while positron emission tomography using synthetic radiolabeled octreotide with radio-nuclide tracers (eg, 68Ga and 18F-dihydroxy-phenyl-alanine) can be useful for detecting cardiac metastases.^23,24^

CHD is a complex and rare disease and should be managed in specialized centers with expertise in this field and with the involvement of a multidisciplinary team of oncologists, cardiologists, endocrinologists, gastroenterologists, and surgeons (colorectal, hepatobiliary, or cardiothoracic surgeons).^5^

Diarrhea is present in up to 75% of patients; it is typically secretory, largely aqueous, and sometimes explosive and life-threatening due to severe dehydration and electrolyte imbalance.^25^

There is no current evidence that medical interventions, such as somatostatin analogues or systemic chemotherapy, are effective in valvular disease,^1^ and bacterial endocarditis prophylaxis is not indicated in patients with CHD.^26^

Bernheim et al found that surgical resection of the underlying tumor retards or prevents CHD, decreases cardiac progression, and improves prognosis.^27,28^ However, cardiac surgery (which primarily consists of valve replacement) is the only definitive treatment option for those with severe CHD, improving both quality of life and overall survival.^4,14^ It should be considered in symptomatic patients with severe valvular involvement and in which CS is well controlled,^29^ with at least 12 months of anticipated postoperative survival from their NET disease. In this setting, indications for tricuspid valve replacement surgery are severe symptomatic tricuspid regurgitation (fatigue and dyspnea leading to edema, ascites) or progressive asymptomatic right ventricular enlargement/dysfunction.^30^ Pulmonary valve replacement is usually performed in case of right ventricular outflow tract obstruction or for treating severe valve regurgitation.^29^ Patent foramen ovale, if present, should also be closed at the time of cardiac valve surgery.

Valve replacement surgery improves patients’ outcome^31,32^ with postoperative median survival ranging from 6 to 11 years.^14^ More recently, in a retrospective study of 195 patients, Connolly et al reported survival rates of 69%, 35%, and 24% at 1, 5, and 10 years, respectively.^29^

Regarding the type of valve prosthesis (biological vs mechanical), the choice is complex and controversial as the literature is limited to small retrospective series or case reports.^3^ The decision should be made on an individual basis, taking into consideration the risk of bleeding, life expectancy (particularly considering the neoplastic disease), and the probability of future therapeutic interventions.^33^

The main disadvantage of mechanical valves is the need for anticoagulation; this can be problematic in patients with advanced neoplasm, due to lability of anticoagulation levels, concomitant secondary liver lesions, or the need for further surgical intervention. Another problem is also the increased risk of thrombosis of mechanical prostheses in the tricuspid position.

Complications can also occur with biological valves, including premature degeneration and dysfunction due to thrombosis^29^ or carcinoid plaque deposition.^34,35^ Newer generation bioprosthetic valves may be more durable.^36^

Balloon valvuloplasty has also been used to treat stenotic valves (pulmonary or tricuspid) when cardiac valve surgery is not indicated (due to performance status or comorbidities). Several cases of percutaneous valve implantation have been reported in patients with NETs showing bioprosthesis dysfunction.^29,37-39^ In these patients, improved functional class and ventricular function have been observed postoperatively, so that it can be consider an interesting therapeutic option in high-risk patients.

Other therapeutic options (eg, medical treatment with somatostatin analogues) or interventions to control tumor spreading (eg, local treatment of secondary lesions of the liver) can improve symptoms of CS but do not affect overall survival.

After establishing the diagnosis of CHD on echocardiography, patients should undergo adequate clinical follow-up. As suggested in the expert statement by Davar et al,^30^ this consists of clinical and echocardiographic evaluation every 6 months in patients with mild CHD, or every 3 months in patients with moderate to severe CHD.

## Conclusion

The present case report, where right heart failure was the symptom that brought our patient to medical attention, underlines the key role of echocardiography in the diagnosis of CHD. The combination of echocardiographic imaging and chromogranin A level provided useful insights, leading to the accurate and undoubted diagnosis, which was subsequently confirmed by histological examination.
